# Evolution and potential function of fibrinogen-like domains across twelve *Drosophila *species

**DOI:** 10.1186/1471-2164-9-260

**Published:** 2008-05-30

**Authors:** Sumit Middha, Xinguo Wang

**Affiliations:** 1 Center for Genomics and Bioinformatics, Indiana University, Bloomington, IN 47405, USA; 2 Bioinformatics Core, Mayo Clinic, Rochester, MN 55905, USA

## Abstract

**Background:**

The fibrinogen-like (FBG) domain consists of approximately 200 amino acid residues, which has high sequence similarity to the C-terminal halves of fibrinogen β and γ chains. Fibrinogen-related proteins (FREPs) containing one or more FBG domains are found universally in vertebrates and invertebrates. In invertebrates, FREPs are involved in immune responses and other aspects of physiology. To understand the complexity of this gene family in *Drosophila*, we analyzed FREPs in twelve *Drosophila *species.

**Results:**

Using the genome data from 12 *Drosophila *species, we identified FBG domains in each species. The results show that the gene numbers in each species vary from 14 genes up to 43 genes. Using sequence profile analysis, we found that FBG domains have high sequence similarity and are highly conserved throughout. By comparison of structure and sequence conservation, some of the FBG domains in *Drosophila melanogaster *are predicted to function in recognition of carbohydrates and their derivatives on the surface of microorganisms in innate immunity.

**Conclusion:**

Sequence and structural analyses show that FREP family across 12 *Drosophila *species contains conserved FBG domains. Expansion of the FREP families in *Drosophila *is mainly accounted by a major expansion of FBG domains.

## Background

In mammals, fibrinogen is composed of six polypeptide chains, two each of the Aα, Bβ and γ chains. Fibrinogen is a soluble plasma protein, which participates in both the cellular phase and the fluid phase of coagulation [1]. The fibrinogen-like (FBG) domain consists of approximately 200 amino acid (aa) residues and has high sequence similarity to the C-terminal halves of fibrinogen β and γ chains. However, the two loops in the fibrinogen γ fragment are shortened by 14 and 7 aa in FBG domain respectively [2,3]. Fibrinogen-related proteins (FREPs) contain one or more FBG domains, which are found universally in vertebrates and invertebrates [2,3]. In mammals, three distinct fibrinogen-related proteins have been identified: ficolin, tenascins, and microfibril-associated protein (MAP) [4-6]. All of these FREPs contain an FBG domain in their C terminus, but differ in their N-terminal regions. Ficolins are the most important molecules that have been studied so far, and they act as pattern recognition receptors to bind pathogens in host innate immunity initiating immune responses [4,7-9]. The FBG domain in ficolins is able to form polymer through collagen O-like triple helices, and is responsible for N-acetylglucosamine (GlcNAc) and other sugar binding activity [4,7-9]. Recent studies have shown that human serum ficolins act as phagocytic receptors on circulating monocytes for microorganism recognition [10].

In invertebrates, several FREPs have been reported in various species, such as tachylectins from the horseshoe crab, *Tachypleus tridentatus *[2], fibrinogen-related proteins (FREP) from the snail, *Biomphalaria glabrata *[11], ficolins from the solitary ascidian, *Halocynthia roretzi *[12], tachylectin-related protein in the sponge, *Suerites domuncula *[13] and aslectin from the mosquito, *Armigeres subalbatus *[14]. All of these FREPs contain a common C-terminal FBG domain. These FREPs likely play an important role in the innate immune response against parasites [2,13,14]. The FBG domain of tachylectin is able to bind GlcNAc [2]. Aslectin can bind GlcNAc and bacteria, and therefore it is likely involved in the antibacterial immune response in mosquitoes [14]. Recently we characterized the FBG domains of FREPs genome-wide in mosquito, *Anopheles gambiae*, and predicted that some of the FBG domains may function by binding to pathogens in host immune response [3]. The FBG domain proteins have been identified and characterized recently with respect to immune response in mosquito [15].

Comparative genome analysis of related species provided a powerful and general approach for identifying functional elements without previous knowledge of function [16,17]. It has substantial power to identify genes, define gene structure, highlight rapid and slow evolutionary change, identify regulatory elements and reveal combinatorial control of gene regulation. Furthermore, it allows identification of all the major differences among the organisms [16-18]. The power is comparable to experimental analysis in terms of sensitivity and precision [17]. With the 11 additional *Drosophila *species genomes sequenced, this extensive sequence resource, encompassing species with well-defined phylogenetic relationships, provides a model system for comparative genomic analyses [19]. In this study, we apply sequence profile analysis and comparative genomics to the wealth of new information from 12 *Drosophila *species genomes to identify FBG domains in FREPs. Provided is an overview of FREP gene family, including sequence alignments, patterns of conservation, phylogenetic relationships and potential function.

## Results and Discussion

### Fibrinogen-related proteins and fibrinogen-like domain in the 12 *Drosophila *species

Fibrinogen-like domains have been well defined in mammals and insects [2,3,20]. In order to select FBG domain seed in *Drosophila melanogaster*, human ficolins were used to BLAST against *D. melanogaster *proteins. The resulting sequences were aligned and the FBG domain of NP_611160 (FBgn0034160) was selected as a seed to perform a BLAST search. Meanwhile, sixty amino acids were used as the minimum length of homology, and protein sequences having 35% or greater amino acid identity were chosen as FREP protein. This search identified 285 FREP proteins in the genome of 12 *Drosophila *species (Table 1). The data showed that the numbers of FREPs in each *Drosophila *species vary from 14 genes up to 43 genes. All the species in *Drosophila *subgenus have more than 20 genes while four of the eight species in *Sophophora *subgenus have more than 20 genes (Table 1). *D. Yakuba *has the least gene number with just 14 genes, and *D. grimshawi *has most gene number, with 43 FREP genes, followed by *D. willistoni *and *D. virilis *with 34 and 33 FREPs respectively (Table 1). Unlike olfactory receptors for which the gene number in each species has been quite stable during evolution [21], the numbers of FREP genes are divergent in *Drosophila *species, suggesting this gene family evolved under relaxed constraints.

**Table 1:** The gene number of fibrinogen-related proteins in 12 *Drosophila *species

Subgenus	Species	Gene Number	Truncated FBG
*Sophophora*	*melanogaster*	17	1
	*simulans*	14	2
	*sechellia*	15	0
	*yakuba*	14	1
	*erecta*	16	1
	*ananassae*	28	2
	*pseudoobscura*	25	2
	*persimilis*	24	3
	*willistoni*	34	6
*Drosophila*	*virilis*	33	2
	*mojavensis*	22	3
	*grimshawi*	43	13

To detect known domain structure in the FREP proteins, the SledgeHMMER program was used to scan the protein sequences [22,23]. The data showed that all the FREP proteins contain at least one FBG domain (Additional file 1). Interestingly, four FREP proteins contain multiple FBG domains, e.g. *dgri _7049*, *dgri _7054 *and *dana _11471 *contain 2 FBG domains and *dwil _15059 *has 3 FBG domains (Additional file 1). Most of the FREP proteins have one FBG domain located in the C-terminus. In these FREP proteins, we found that the majority contain a full length FBG domain composed of approximately 200 amino acids, which is similar to the full length FBG domains in human and mosquitoes [2,3,20]. However, some of the FREPs contain a truncated FBG domain (Table 1 and Additional file 1). This could be caused by sequencing error or automated annotation [3,22]. Besides FBG domains in FREP proteins, we also found that 14 of FREP proteins contain other known domains; detailed information is available in Additional file 1.

### Phylogenetic relationship of fibrinogen-like domains and chromosomal location of fibrinogen-related proteins

**Figure 1:**
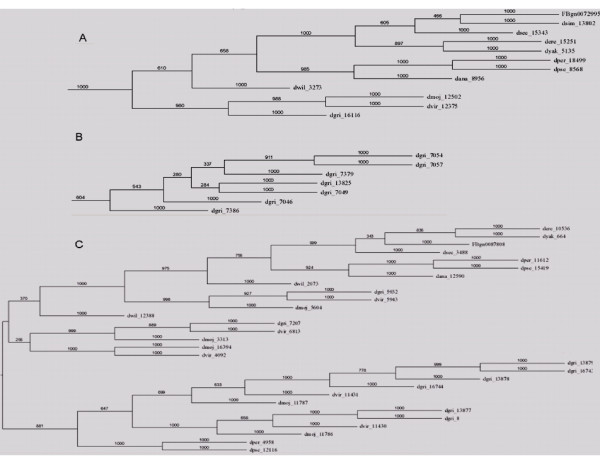
**Selected clusters of phylogenetic tree of the FBG domains in the 12 Drosophila species**. The seed sequence used for constructing the tree was the multiple sequence alignment of FBG domains that excluded truncated FBG domains. Bootstrap was applied to the data. Protein distance was calculated using the Jones-Taylor-Thornton model of change between amino acids and a Hidden Markov Model (HMM) method of inferring different rates of evolution at different amino acid positions. Neighbor-joining was applied to produce the tree. The FBG domains of each FREP are denoted by their gene name or GLEANR gene ID. A. A cluster composed of FBG domains from multiple species (Group D in supplement fig. 2). B. A cluster composed of FBG domains from a single species (*D. grmshawii*, Group K in supplement fig. 2). C. Majority of FBG domains from *D. virilis, D. mojavensis *and *D. grimshawi *are clustered together (Group G in supplement fig. 2).

To analyze the evolutionary history of FBG domains in the FREP family, a phylogenetic tree was constructed with the alignments of the conserved FBG domains using Neighbor Joining. The striking pattern in this tree is that of FBG domains from different species being grouped together, indicating that they are orthologous genes (Fig. 1A, Table 2 and Additional file 2). This suggests that these FBG domains are stable across *Drosophila *species during evolution. We also found that some clusters are composed of FBG domains only from one species, such as *D. grimshawi *(Fig. 1B and Table 2), suggesting that gene expansion occurred in *D. grimshawi *after divergence of this species from the others. The FBG domains from *D. willistoni *formed two separate branches far apart in the tree (Additional file 2). Furthermore, some clusters are composed of FBG domains from a few *Drosophila *species instead of all, e.g, the majority of FBG domains from *D. virilis, D. mojavensis *and *D. grimshawi *are clustered together, and FBG domains from *D. persimilis *and *D. pseudoobscura *form a cluster as well (Fig. 1C, Table 2 and Additional file 2). Interestingly, in the evolutionary tree, for *D. virilis, D. mojavensis *and *D. grimshawi *of *Drosophila *subgenus, the FBG domains are clustered together and far apart from *Sophophora *subgenus, suggesting that the gene divergence had occurred after divergence of these three species from the others.

**Table 2:** Numbers of fibrinogen-related proteins for 14 clades in each *Drosophila *species

Species\Clade	A	B	C	D	E	F	G	H	I	J	K	L	M	N
*melanogaster*	2	1	1	1	0	2	1	2	0	3	0	0	1	2
*simulans*	2	1	1	1	1	1	0	1	0	2	0	0	1	1
*sechellia*	2	1	1	1	1	2	1	1	0	2	0	0	1	1
*yakuba*	2	1	1	1	0	2	1	0	0	2	0	0	1	1
*erecta*	2	1	1	1	1	1	1	1	0	3	0	1	1	1
*ananassae*	2	2	0	1	1	7	4	0	1	2	0	1	1	1
*pseudoobscura*	2	0	2	1	0	7	2	1	0	3	0	1	1	1
*persimilis*	2	0	1	1	0	8	2	1	0	4	0	1	0	1
*willistoni*	3	1	8	1	0	4	2	1	1	2	0	0	1	1
*virilis*	2	2	1	1	3	1	6	3	0	7	1	0	1	1
*mojavensis*	2	1	0	1	2	1	6	0	1	2	1	0	1	0
*grimshawi*	1	2	0	1	3	0	8	2	0	2	7	0	1	1

**Figure 2:**
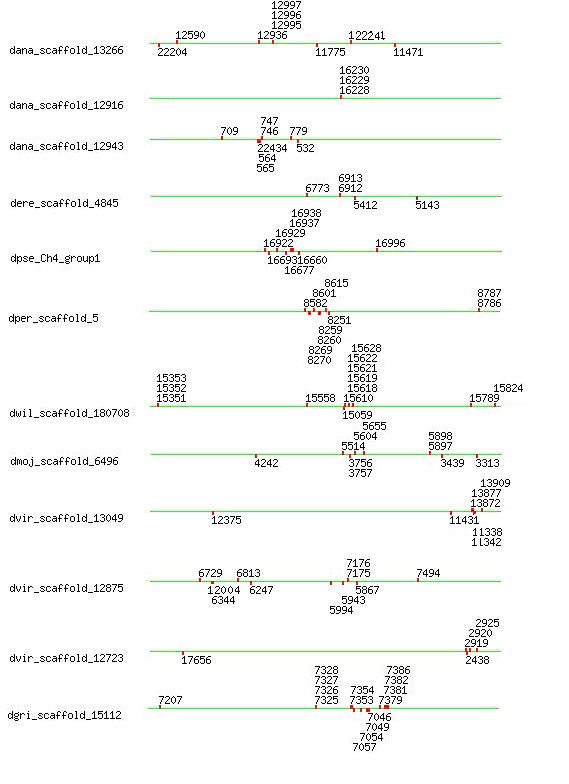
**Scaffold location of selected FREPs in the *Drosophila *species**. Gene locations for the FREP family were retrieved from the AAA database. Scaffold was named using single letter from genus plus the first three letters from subgenus to differentiate species. The scaffold is represented with a green line, which is not scaled. The relative location for each gene was shown on the scaffold using their GLEANR gene ID.

To understand the evolutionary history of this gene family across closely related species, we also compared the correlations between chromosomal locations of FREP and sequence similarities of FBG domains among the family members. Gene locations for the FREP family have been retrieved from the AAA database [24]. None of the FREP genes were observed on the dot chromosome, which is chromosome 4 in *D. melanogaster*. Some of FREP genes are arrayed in tandem and form clusters (Fig. 2), especially the clusters in the *Drosophila *subgenus of *D. mojavensis*, *D. virilis *and *D. grimshawi*, and the similar pattern was also observed in *Sophophora *subgenus, including *D. pseudoobscura*, *D. persimilis *and *D. wilistoni *(Fig. 2). If the number of FBG domains increased mainly by tandem duplication, we would expect the domains which are physically clustered in the genome to form a monophyletic group. By examining the relationships between phyletic pattern and chromosomal location of the FBG domains, this pattern was only identified in *D. wilistoni*, which is that the genes clustered on a single *D. wilistoni *scaffold occurred to be in close-by branch in the phylogenetic tree (Fig. 2 and Additional file 2). The vast majority of FBG domains closely located in the genome are scattered in different clusters in the phylogenetic tree (Fig. 2 and Additional file 2). This suggests that a dynamic history for the FBG domains likely involved shuffling among chromosomes. The predicted role, for at least a subset of these FBG domains, is in carbohydrate sensing (see below). This expansion in the *Drosophila *genome may have been a response to the diversity of carbohydrates encountered, resulting in the utilization of numerous FBG domain variations in order to recognize a broad range of different carbohydrates.

### Conserved structure of the FBG domains and their potential function

**Figure 3:**
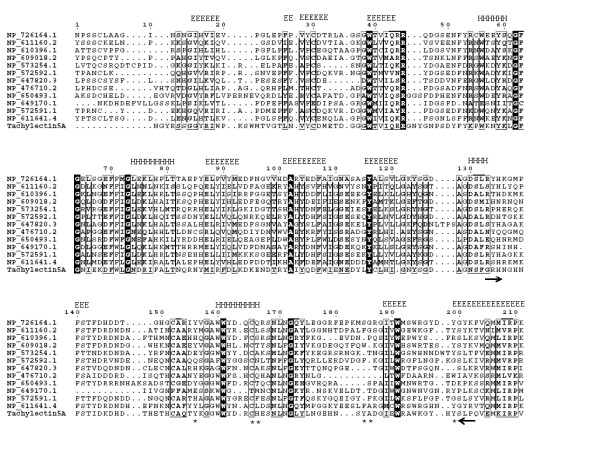
**Multiple sequence alignment of a representative set of the FBG domains in *D. melanoganster***. Multiple sequence alignment was constructed using T-Coffee program. The 100% consensus sequence was boxed with black in the alignment. The PHD secondary structure is shown above the alignment with H representing an α-helix and E representing a β-strand. The sequences are denoted by their gene names in GenBank. The domain P is indicated between two arrows. The amino acids involved in forming binding pocket were shown in star.

In order to construct an optimal multiple alignment of the FBG domain, we first aligned selected sequences from *D. melanogaster *with the T_Coffee program [25,26] (Fig. 3). Multiple alignment of the FBG domain sequences showed that FBG domains are highly similar throughout. Using the multiple alignments of the FBG domains as queries, the secondary structure was predicted with the PHD program [27], which showed that the FBG domains have a highly conserved structure profile (Fig. 3). To further compare the predicted secondary structure of the FBG domains with known structures, we found that the FBG domain is structurally related to the FBG domains of human ficolin and TL5A in the protein data bank (PDB) [20,28]. The FBG domains of human ficolin and TL5A comprise of a central and larger domain B and a relatively smaller domain P [20,28]. The domain B is predominantly built up by a twisted seven stranded anti-parallel β-sheet (strands β3-β7, β9 and β12) and helices α4 and α5 functioning as the backbone. The domain P possesses only a few short elements of secondary structure, and comprises the major functional site forming a binding pocket [20,24]. The predicted secondary structures of the FBG domains in the FREP gene family approximately correspond to the domain architectures of FBG domains in human ficolin and TL5A (Fig. 3). The β-sheets and α-helices in the predicted structure of the FBG domain are highly conserved with the corresponding structures in TL5A, especially in the domain B (Fig. 3). For example, the central strand β12, which extends the C terminus of domain P back to domain B and brings both polypeptide termini in close proximity, was also seen in *Drosophila *FBG domains (Fig. 3). This suggests that the FBG domain architecture is conserved among human, horseshoe crab and *D. melanogaster*. The projection of some of the highly conserved domains that form the ligand-binding pocket suggests that the core structure of the ligand-binding pocket is also likely to be conserved across these FBG domains (Fig. 3). These observations imply that the FBG domains are most likely to function as receptors for carbohydrates or their derivatives. Beyond the common core, FBG domains also show great diversity in terms of the insertions and deletions among the conserved domains, for example, NP_476710 loses a conserved domain due to deletion and NP_647820 has a short insertion located in the loop region (Fig. 3). By comparison of amino acids in the FBG domains of FREP corresponding to the P domain binding site in TL5A, we found that the domain architectures of these FBG domains have considerable diversity that is incorporated into a shared basic architectural blueprint (Fig. 3). In invertebrates, several FREP proteins have been reported to play an important role in innate immunity and in particular in the recognition of parasites [2,13,14]. Aslectin can be upregulated by bacterial challenge and is able to bind GlcNAc and bacteria [14]. The FBG domain of TL5A can form a ligand-binding pocket specifically recognizing the acetyl-group in eliciting an immune response [20]. These data suggest that the FBG domains of FREPs in *D. melanogaster *probably function in recognizing carbohydrate moieties in innate immunity. These were further supported by microarray analysis in *melanogaster *from GEO profile [29]. NP_611160 was upregulated by virus infection, fugal infection and injury [30,31]. NP_649170 was upregulated in cell line following LPS or *E. coli *infection [31]. Phenobarbital is a xenobiotic that triggers a defense response, inducing genes that encode key detoxification enzymes. DHR96 is a xenobiotic receptor that controls metabolic and stress-response genes [32]. NP_573254 is down-regulated following Phenobarbital treatment, but is not affected by DHR96. However, NP_647820 and NP_723894 are regulated by DHR96 receptor [32]. These suggest that FREP genes play important roles in both innate immunity and physiology.

## Conclusion

The data demonstrated that the number of FREP genes in each *Drosophila *species vary from 14 genes up to 43 genes. Some of the FBG domains from different species are grouped together in the phylogenetic tree, indicating that they are orthologous genes. This suggests that these FBG domains are stable across *Drosophila *species in evolutionary process. We also found that some clusters in the tree are composed of FBG domains only from one species or a few species instead of all 12 *Drosophila *species. By comparison of phylogenetic tree and chromosomal location of FREPs, we found that expansion of the FREP families in *Drosophila *is mainly accounted for by a major expansion of FBG domains, and both tandem duplication and shuffling are involved in gene expansion of FREPs. The results from sequence and structural analyses imply that FBG domains are highly similar across 12 *Drosophila *species. Some of the FBG domains in *Drosophila melanogaster *are predicted to function in recognition of carbohydrates and their derivatives on the surface of microorganisms in innate immunity.

## Methods

### Database searching and sequence retrieving for fibrinogen-related protein

Fibrinogen-like domain seed sequence was used to align with the *Drosophila *proteome sequences using the BLAST search. Flybase version 5.1 data was used for *D. melanogaster *while Comparative Annotation Freeze 1 (CAF1) data was used for the other 11 *Drosophila *species [33,34]. The 11 *Drosophila *species other than *D. melanogaster *are *D. simulans, D. sechellia, D. yakuba, D. erecta, D. ananassae, D. pseudoobscura, D. persimilis, D. willistoni, D. mojavensis, D. virilis*, and *D. grimshawi*. The genome sequences are available from the Assembly, Alignment and Annotation of the new 12 related *Drosophila *genomes [24]. The genus name will be omitted in results and discussion. Translation sequences of GLEANR gene models we used for the 11 *Drosophila *species were downloaded from AAA datasets on January 2007. The resulting protein sequences from the first search were used to iterate the search and retrieve any left out FREPs. Sixty amino acids was used as a minimum length of the match along with 35% homology in order to add a protein to the list of FREPs. The list was manually checked and a non-redundant set of protein sequences was obtained.

### Domain identification in *Drosophila *fibrinogen-related proteins

SledgeHMMER was used to carry out batch searching of the current Pfam database (version 20.0) using the 'hmmpfam' program [22,23].

### Genome location of fibrinogen-related proteins

Perl scripts were used to extract the chromosome location for each gene (or scaffold if chromosome information is not available) and show the distribution of FREPs in the respective genomes of the 12 *Drosophila *species.

### Multiple sequence alignment and phylogenetic analysis

Multiple sequence alignment was performed using the ClustalW for large data set or T_Coffee program [25,26,35]. Multiple sequence alignment was visualized using ESPript [36]. For phylogenetic tree construction, the seed sequence was the multiple sequence alignment of FBG domains that excluded truncated FBG domains. Bootstrap was applied to the data. Protein distance was calculated using the Jones-Taylor-Thornton model of change between amino acids and a Hidden Markov Model (HMM) method of inferring different rates of evolution at different amino acid positions. Neighbor-joining was applied to produce the tree. Phylogenetic analysis was carried out with the package from PHYLIP [37], the consensus tree was drawn using the consense program and visualized using the drawgram tool in this package.

### Secondary structure prediction

Secondary structure prediction was produced with the PHD program [27], with multiple sequence alignment of FBG domains. The structure data of TL5A was obtained from protein data bank (PBD) [38].

### Microarray data retrieval

In order to find gene expression profiles in microarray analysis, GEO profiles from NCBI [29] were searched for each member of FREPs from *D. melanogaster*.

## Authors' contributions

SM carried out the database survey. He identified and analyzed the FBG domains, and prepared the manuscript. XW conceived the study and contributed to the preparation of the manuscript. All authors read and approved the final manuscript.

## Supplementary Material

Additional file 1 **The distribution of fibrinogen-like domains in fibrinogen-related proteins**. SledgeHMMER was used to carry out batch searching of the current Pfam database (version 20.0) to identify known domains using the 'hmmpfam' program. Gene ID was shown in the left side of each gene. The domains were indicated in the red. The domain name was shown below the domain detected by SledgeHMMER. Click here for file

Additional file 2 **Phylogenetic tree of the fibrinogen-like domains**. The seed sequence used for constructing the tree was the multiple sequence alignment of FBG domains that excluded truncated FBG domains. Bootstrap was applied to the data. Protein distance was calculated using the Jones-Taylor-Thornton model of change between amino acids and a Hidden Markov Model (HMM) method of inferring different rates of evolution at different amino acid positions. Neighbor-joining was applied to produce the tree. The FBG domains of each FREP are denoted by their gene name or GLEANR gene ID. Click here for file
