# Supplementary figures and images for: Evolution and potential function of fibrinogen-like domains across twelve Drosophila species
Source: BMC Genomics. 2008 May 30;9:260. doi: 10.1186/1471-2164-9-260 (PMC2429915; doi:10.1186/1471-2164-9-260)

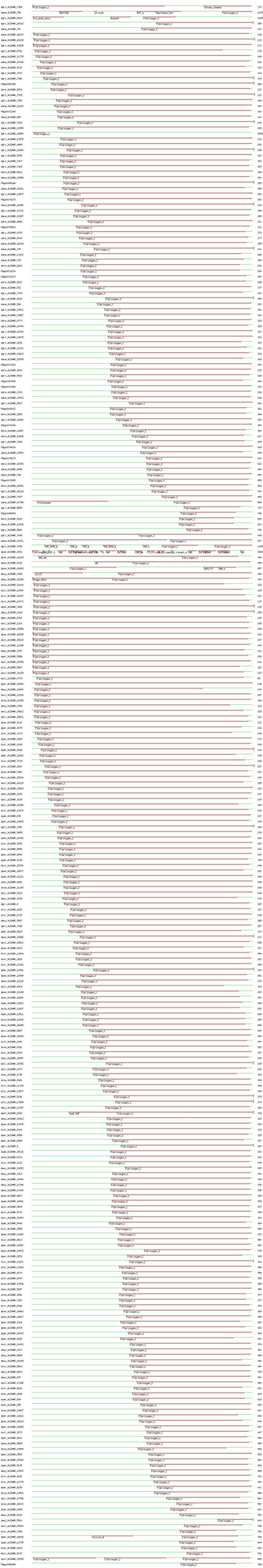

Supplement: Additional file 1 — The distribution of fibrinogen-like domains in fibrinogen-related proteins. SledgeHMMER was used to carry out batch searching of the current Pfam database (version 20.0) to identify known domains using the 'hmmpfam' program. Gene ID was shown in the left side of each gene. The domains were indicated in the red. The domain name was shown below the domain detected by SledgeHMMER. [file 1471-2164-9-260-S1.jpeg]

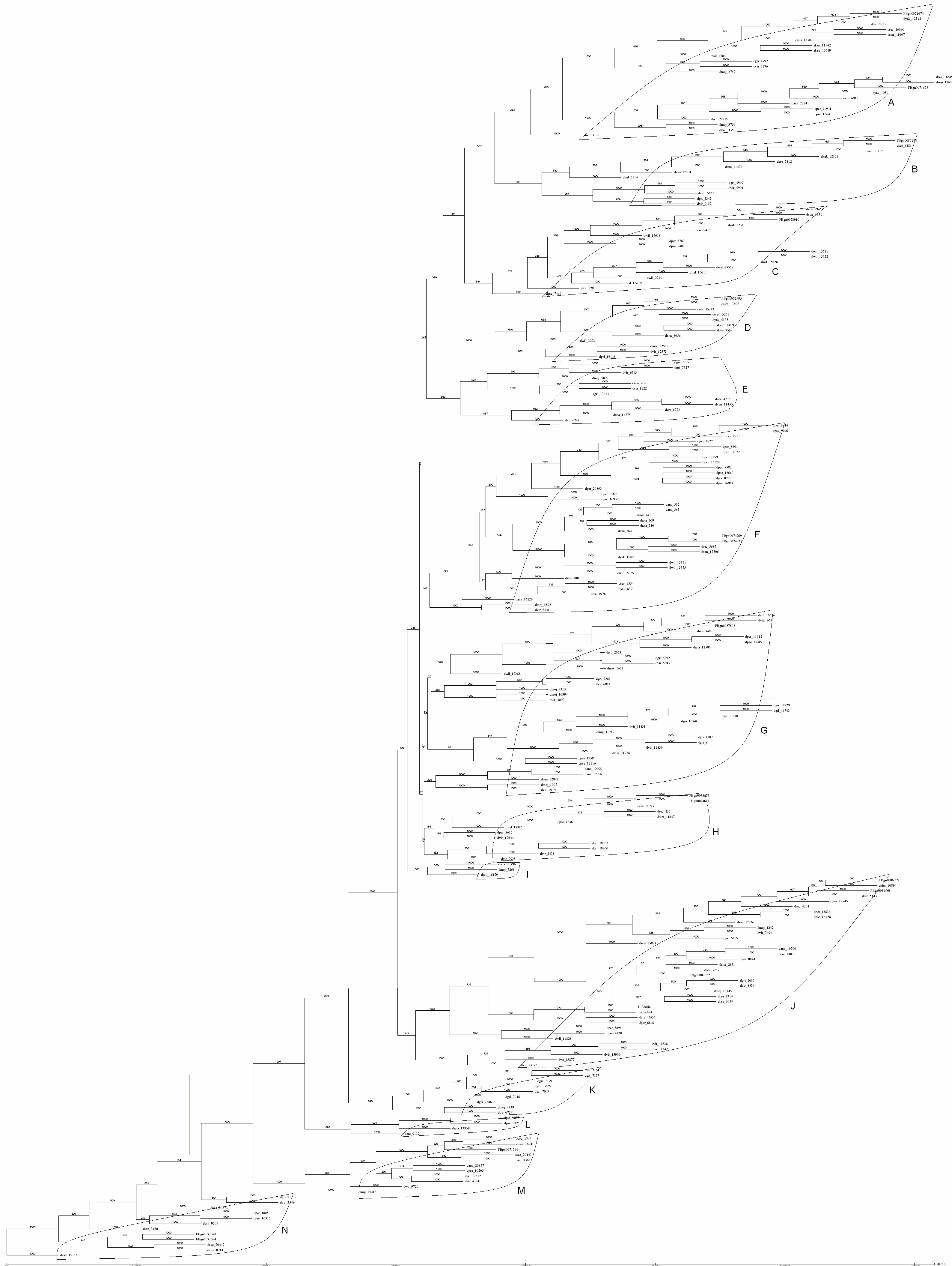

0 100 200 300 400 500 600 700 800 900 1000

Supplement: Additional file 2 — Phylogenetic tree of the fibrinogen-like domains. The seed sequence used for constructing the tree was the multiple sequence alignment of FBG domains that excluded truncated FBG domains. Bootstrap was applied to the data. Protein distance was calculated using the Jones-Taylor-Thornton model of change between amino acids and a Hidden Markov Model (HMM) method of inferring different rates of evolution at different amino acid positions. Neighbor-joining was applied to produce the tree. The FBG domains of each FREP are denoted by their gene name or GLEANR gene ID. [file 1471-2164-9-260-S2.pdf]
